# Intake of Vitamin D in Patients with Multiple Sclerosis in the Valencian Region and Its Possible Relationship with the Pathogenesis of the Disease

**DOI:** 10.3390/life11121380

**Published:** 2021-12-10

**Authors:** Jose Enrique de la Rubia Ortí, María Faus García, Eraci Drehmer, Esther Navarro-Illana, Julia Casani-Cubel, Belén Proaño, Claudia Emmanuela Sanchis-Sanchis, Juan Doménech Escrivá

**Affiliations:** 1 Department Nursing, Catholic University San Vicente Mártir, 46001 València, Spain; maria.faus@ucv.es (M.F.G.); esther.navarro@ucv.es (E.N.-I.); juandoes1984@gmail.com (J.D.E.); 2 Department of Basic Sciences, Catholic University of Valencia San Vicente Mártir, 46900 Torrente, Spain; eraci.drehmer@ucv.es; 3 Doctoral Degree School, Health Sciences, Catholic University of Valencia San Vicente Mártir, 46001 València, Spain; juliacasani@mail.ucv.es (J.C.-C.); beprool@mail.ucv.es (B.P.)

**Keywords:** multiple sclerosis, vitamin D, interleukin-6

## Abstract

(1) Background: Multiple sclerosis (MS) is a neurodegenerative disease characterized by pronounced inflammation. Interleukin 6 (IL-6) is an accurate marker for the state of inflammation, due to the high levels of this cytokine linked to the pathogenesis of the disease. These IL-6 levels could be lowered with an adequate dietary intake of vitamin D. The objective of the study was to determine the level of vitamin D ingested in a sample of patients with MS in the Valencian region (Spain), to establish the vitamin sources, and the possible link between the intake of vitamin D and the pathogenesis of the disease through a relationship with the level of IL-6. (2) Materials and Methods: A descriptive pilot study was carried out with 39 patients with MS in the Valencian region. The dietary-nutritional anamnesis was gained through the food frequency questionnaire (FFQ) and a food diary. Diet and eating habits were analyzed through the Easy Diet (version: 2.0.1)—Consultation Management Program^®^ software, and IL-6 levels in blood by ELISA technique. (3) Results: The results show a low intake of vitamin D, which is significantly and negatively related to the intake of proteins of vegetable origin, which are consumed in less quantity than proteins of animal origin, and significantly and negatively related with the high blood levels of IL-6, possibly as a consequence of the high intake of fats, mainly unsaturated. (4) Conclusions: MS patients in the Valencian region ingest little vitamin D related to low intake of vegetable protein, which would explain the high levels of IL-6 linked to the high intake of mainly saturated fats.

## 1. Introduction

Multiple sclerosis (MS) is a chronic, inflammatory, autoimmune, neurodegenerative and demyelinating disease of the central nervous system (CNS). This neuronal damage is mainly based on high oxidative stress and inflammation in the CNS [1].

Due to the inflammatory nature of the disease, several inflammatory markers have been linked to MS. Among these, interleukin 6 (IL-6), with significantly high levels in patients with MS [2], seems to be directly related to demyelination, since an increase in IL-6 receptors has been seen in CD4+ T cells of patients with MS, involved in the pathogenesis of the disease [3].

Regarding the protective factors of the pathology, the activity of vitamin D could be especially relevant, as it has immunomodulatory properties [4]. Moreover, high levels of vitamin D decrease the risk of developing the disease [5,6], and its supplementation in MS patients achieves anti-inflammatory and immunomodulatory effects [7]. It is interesting to highlight how this immunomodulation is carried out partly through the regulation of different interleukins, such as IL-6, since it has been seen that vitamin D is able to reduce its production and modulate its activity [8], potentially decreasing its pro-inflammatory effects and improving MS prognosis [9].

The main source of vitamin D is related to sun exposure; as such, Spanish populations in the Mediterranean basin, including those living in the Valencian region, should not be deficient due to the large number of hours of sunlight per year [10]. However, numerous cases of disorders such as rickets or severe vitamin D deficiencies are reported in Spain [11], among which are those produced in Valencia [12]. This is why the intake of the vitamin through food may be particularly relevant, and a deficiency of vitamin D from food has been linked to a higher prevalence of other immune-mediated diseases such as psoriasis [13].

The best nutritional sources for vitamin D are blue fish (salmon, sardine, tuna, herring or mackerel), cod liver oil, red meat, eggs, mushrooms and fortified foods (milk, cereals, cheese and orange juice) [14,15]. However, the influence of the different vitamin D rich foods on the progress of the disease is different, as it has been found that diets rich in foods of vegetable origin show a decrease in lesions in magnetic resonance imaging, an improvement in fatigue, depression and quality of life, and a reduction of inflammatory markers, oxidative stress and autophagy [16,17]. On the contrary, the intake of foods rich in protein of animal origin has been related to a worse prognosis of the disease due to the high content of saturated fats that these nutrients also contain [17,18]. As a matter of fact, given the fat-soluble nature of vitamin D, it is more efficiently absorbed when consumed together with fats [19]. The nature of the fats seems important, since a diet rich in saturated fat has been linked to a poor absorption of vitamin D [20], as well as a worse prognosis of the disease [18], unlike a diet rich in vegetable fats [21]. This may be due to the inflammatory nature of these saturated fats, linked to increased inflammatory blood markers [22], and, in particular, IL-6 [23].

For these reasons, the aim of this study is, on the one hand, to determine the level of vitamin D ingested by a population sample of patients in the Valencian region suffering from MS, and its nutritional source, and, on the other hand, to establish the possible link between ingestion of vitamin D and the pathogenesis of the disease through a possible relationship with IL-6 blood level.

## 2. Materials and Methods

A pilot, descriptive, cross-sectional, analytical and quantitative observational study was carried out.

### 2.1. Sample

**Figure 1:**
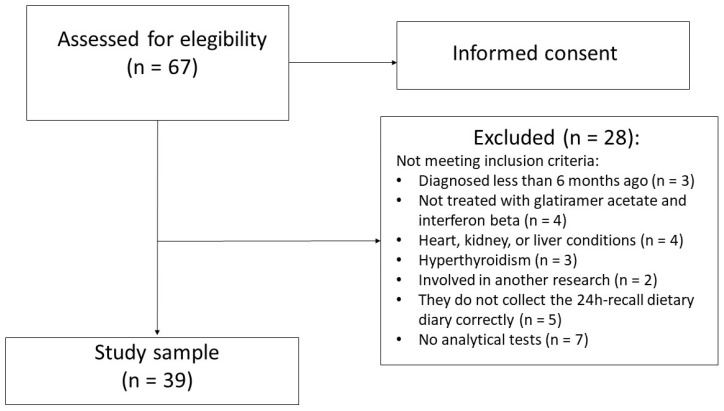
Flow chart of MS patients’ selection for the descriptive, cross-sectional, analytical and quantitative observational study.

To obtain the population sample of the study, the main MS associations in the Valencian region (Spain) were informed, which in turn informed their members. Sixty-seven MS patients diagnosed using the McDonald criteria (2001) [24] were interested in participating in the study and, after signing the informed consent, the following inclusion criteria were applied: patients over 18 years of age diagnosed with MS at least 6 months prior to the study, and treated with glatiramer acetate and interferon beta. The exclusion criteria were: pregnant or lactating women, patients with a tracheostomy, with stomas or short bowel syndrome, suffering from dementia, alcohol or drug abuse, myocardial infarction, cardiac failure, arrhythmias, angina or other cardiac pathologies, patients with renal conditions and creatinine levels twice as high as normal, patients with elevated liver markers three times higher than normal or suffering from chronic liver disease, patients with chronic metabolic disorders, with acromegaly, with polycystic ovarian syndrome, and MS patients participating in other studies related to some type of experimental intervention. These criteria were assessed from the medical records requested from the patients who voluntarily agreed to participate. Once the selection criteria were applied, 28 patients were excluded and the final sample consisted of 39 patients with relapsing–remitting MS and secondary progressive MS defined by Lublin et al. (2013) [25] (Figure 1).

### 2.2. Statistical Analysis

Data were analyzed with SPSS v.23 (IBM Corporation, Armonk, NY, USA, EE. UU.). The Kolmogorov–Smirnov test was used for normal distribution of the variables. This analysis demonstrated non-normal distribution of all the variables studied. Therefore, a two-tailed Spearman′s test was performed for the correlation analysis. A *p*-value below 0.05 was considered significant. Data are presented as mean ± standard deviation, and as median and interquartile range (IQR), or the number of patients and percentage. A multiple linear regression analysis was performed for IL-6 levels as the outcome variable with the variables: age, MS type, expanded disability status scale (EDSS), body mass index (BMI), ingestion of Vitamin D, vegetable protein and animal protein as the predictor variables.

### 2.3. Measurements

A description of dietary habits was expected from each patient, and at the beginning of the study, participants completed a food frequency questionnaire (FFQ) [26] to determine the frequency of weekly and monthly food consumption in order to obtain a dietary/nutritional history. Simultaneously, each participant recorded their food consumption over seven days. For that purpose, they were provided with a series of instructions on weight and portion measurements, as well as cooking methods. A nutritional calibration of the macro and micronutrients consumed by each individual was performed with the collected data by using the EasyDiet^®^ software, version: 2.0.1 [27]. The obtained results were used to calculate the average nutritional intake of each nutrient. Additionally, to assess whether the diet was adequate, the dietary reference intake (DRI) was obtained from guidelines such as the reference intake for the Spanish population [28] and from the consensus of the Spanish Society of Community Nutrition (SENC) [29].

The measurements were obtained by a level 3 anthropometrist certified by the International Society for the Advancement of Kinanthropometry (ISAK), in line with the protocol established by the society [30]. The validated anthropometric material used was the following: portable clinical SECA scale model 874 (150–200 kg capacity, 100 g precision) and a SECA height rod, model 220 (0.1 cm precision) both of Hammer Steindamm 3-25 22089 Hamburg, Germany; metal, inextensible and narrow anthropometric tape, model Lufkin W606PM (0.2 mm precision); a mechanical skin fold caliper, by Holtain LTD, Crymych, UK (0.2 mm precision, measurement range 0–48 mm); a Holtain bicondylar pachymeter to measure the diameter of small bones (1 mm precision, measuring range 0–140 mm) and a dermographic pencil to mark anatomical points. The variables measured were body weight, waist and hip circumference, and triceps, subscapular, supraspinal and abdominal folds. Measurements were carried out twice, but a third measurement was obtained when the difference between the first two measurements was greater than 5% for the folds and 1% for the other measurements.

Functional disability was also measured with the EDSS [31], which is an ordinal scale based on a neurological examination of the eight functional systems (pyramidal, cerebellar, brainstem, mental, sensory, visual, bowel and bladder), alongside assessing walking capacity, which provides a disability index between 0–10, 0 being normal health and 10 understood as death by MS.

In order to analyze IL-6 levels in blood, fasting blood samples were obtained from all patients from their antecubital vein at 11 a.m. Blood was collected in blood bottles from BD Vacutainer Plus ref. 367815 (Madrid, Spain). The tubes were then left at room temperature for 30 min for the blood to clot. To separate the clotted part, the samples were centrifuged at 4000 rpm for 10 min in a refrigerated centrifuge. After centrifugation, blood serum was transferred to 0.5 mL aliquots, which were frozen and stored at −80 °C. Finally, the aliquots were thawed after 24 h, and the concentration of IL-6 was determined by enzyme-linked immunosorbent assay (ELISA) (R & D Systems) (Minneapolis, MN, USA). This assay uses the quantitative sandwich enzyme immunoassay technique, where a monoclonal antibody specific for IL-6 has been pre-coated onto a microplate; standards, control, and samples are pipetted into the wells, and any IL-6 present is bound by the immobilized antibody. After rinsing unbound substances, if any, an enzyme-linked polyclonal antibody specific for rat IL-6 is added to the wells. After a wash to eliminate any unbound antibody-enzyme reagent, a substrate solution is added to the wells. Following this enzyme reaction, a blue product is obtained, and it turns yellow when the stop solution is added. The intensity of the color measured develops in proportion to the amount of IL-6 bound in the initial step. The sample values are then read off the standard curve.

### 2.4. Ethical Concerns

The study was carried out according to the ethical principles for medical research outlined in the Declaration of Helsinki [32], prior approval of the protocol by the Human Research Committee of the University of Valencia, from the Ethics Committee in Experimental Research (procedure number H1512345043343). All study participants, once informed of the nature of the study throughout a patient information sheet, were also provided with an informed consent form that they signed in order to participate in the study.

## 3. Results

After applying the selection criteria in the Material and Methods section, and after the withdrawal of some participants due to different reasons, we had a final sample of 39 patients with MS, whose demographic characteristics are shown in Table 1. Twenty-three participants were women (58.97%) and 16 were men (41.03%). As per the type of MS, 28 patients had relapsing–remitting MS (71.79%) and 11 had secondary progressive MS (28.2%).

**Figure 2:**
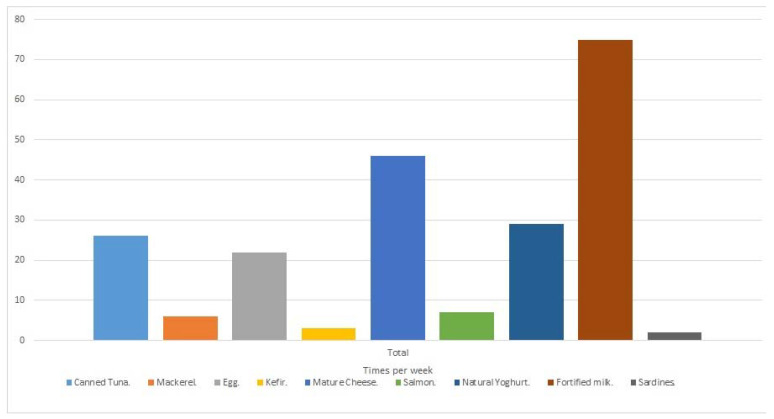
Weekly consumption of the main vitamin D rich foods by patients with multiple sclerosis (MS).

In relation to the estimated levels of vitamin D ingested by the patients, from the food consumption patient register, the mean value was 4.5 ± 2.7 µg, with a median (IQR) of 4.1 (2.6–5.9) µg. These values were below the recommended values (between 5–10 µg/day, depending on age). In order to determine the main sources of vitamin D (specifically Vitamin D3 or cholecalciferol as it is fundamentally of animal origin), a dietary analysis of the eating habits of the participants was carried out, which showed that patients mainly consumed vitamin D fortified milk (approximately five times a week), cured cheese (three times a week) and fortified yoghurts (twice a week). It was also seen that the participants consumed blue fish, such as canned tuna, to a lesser extent (about twice a week), while salmon, mackerel and sardines, rich in vitamin D, were consumed very little (less than once a week) (Figure 2).

**Figure 3:**
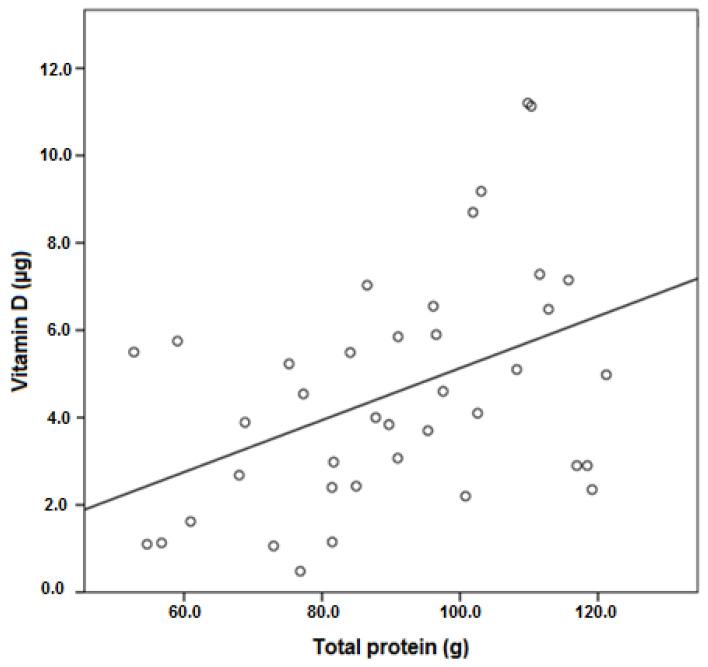
Spearman’s correlation (r_s_ = 0.429; *p* = 0.006) between vitamin D and protein consumption by the MS patients of the study group.

Regarding the associations of vitamin D consumption with the main macronutrients (carbohydrates, proteins and fats), Spearman’s correlation showed only one significant association of vitamin D with protein ingestion (Figure 3). It was a positive relationship (r_s_ = 0.429; *p* = 0.006) indicating that the patients who consumed more protein were also the ones who ingested more vitamin D.

**Figure 4:**
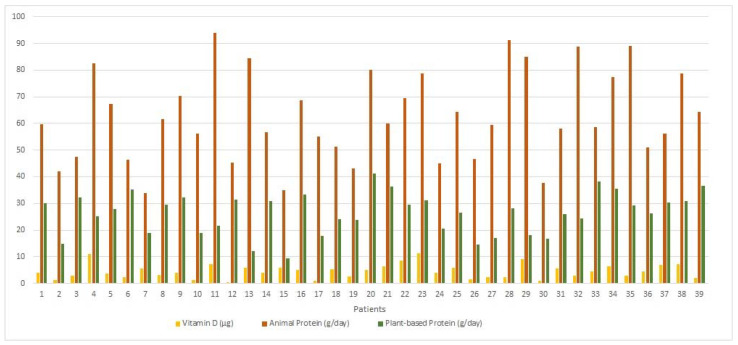
Daily patterns of vitamin D, animal protein and vegetable protein ingestion in the MS patients of the study group. Regarding fats, necessary for vitamin absorption.

After analyzing the participants’ food consumption, it could also be seen that the protein ingested was mainly of animal origin, with a mean of 62.57 ± 16.74 g/day and median of 64.3 (53.2–79.4) g/day, whereas protein of vegetable origin was 26.31 ± 7.72 g/day, with median 28.1 (19.7–31.3) g/day (Figure 4).

**Figure 5:**
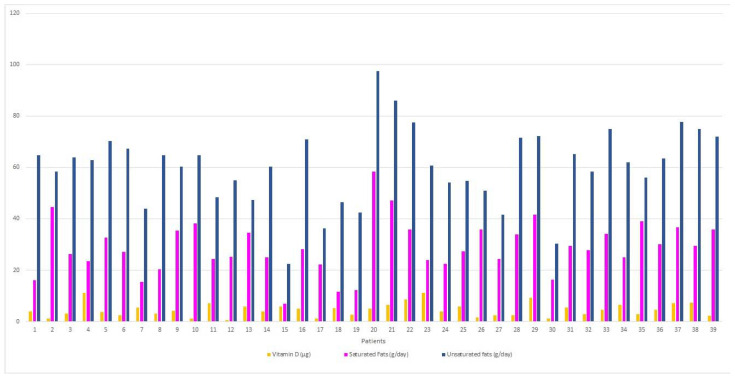
Daily pattern of vitamin D, saturated and unsaturated fats ingestion in patients with MS of the study group.

Figure 5 shows that the participants consumed saturated fats with a mean of 28.84 ± 10.29 g/day and median of 27.3 (22.9–35.9) g/day, which represented a percentage of 10.3% of the total fat whereas the mean for unsaturated fats was 60.34 ± 14.90 g/day. Polyunsaturated fat consumption was lower than monounsaturated, with median of 26.3 (20.2–30.8) g/day and 34.1 (29.5–40.5) g/day. The relationship of saturated and unsaturated fat consumption for each patient, also considering vitamin D, can be seen in Figure 5.

**Figure 6:**
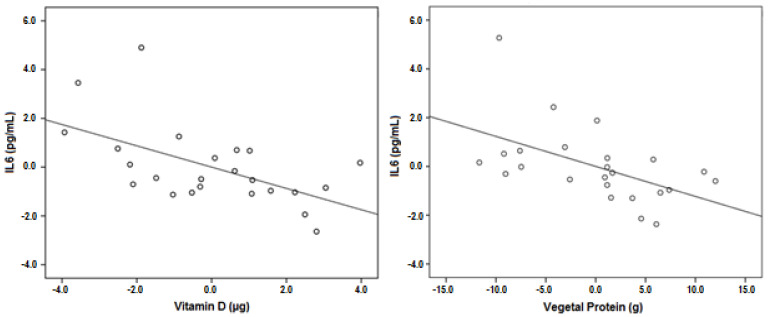
Partial regression plots for IL-6 blood levels in relation to vitamin D (b = −0.437; *p* = 0.014) and vegetable protein ingestion (b = −0.123; *p* = 0.026) in MS patients of the study group.

On the other hand, the mean IL-6 blood levels were 3.51 ± 3.26 pg/mL, with median of 2.5 (1.6–3.6) pg/mL, well above the normal levels, which are 1.4 pg/mL in healthy individuals. Furthermore, the model obtained by multiple linear regression for IL-6 levels with age, MS type, EDSS, BMI, ingestion of Vitamin D, animal and vegetable protein as predictor variables, was statically significant (F_16,23_ = 2.974, *p* = 0.034) with *R*^2^ = 0.565. The regression coefficients (b) and *p*-values can be seen in Table 2. It can also be seen that there was no collinearity since none of the variance inflation factors (VIF) were higher than 2. The model showed a significant effect of Vitamin D and vegetable protein ingestion on IL-6 levels. These were negative associations (as shown by the negative coefficients) in which, per each microgram (ug) of ingested vitamin D, IL-6 decreases in 0.437 ug/mL; and per each gram of ingested vegetable protein, IL-6 levels decrease 0.123 ug/mL. These relationships are represented in Figure 6.

**Figure 7:**
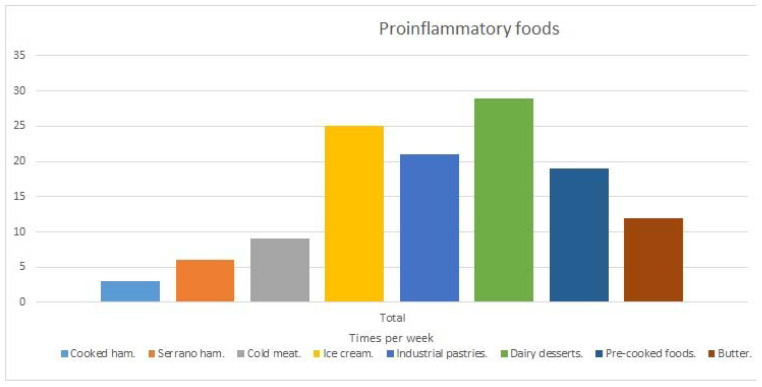
Description of eating habits of the participants with MS, related to saturated fat foods and of pro-inflammatory foods.

Finally, given the influence of low vitamin D intake on high levels of the pro-inflammatory cytokine IL-6, the consumption of foods considered to be pro-inflammatory and with little vitamin D intake was analyzed further. The number of times a week the participants ate processed foods was also analyzed, showing the high intake of industrial dairy desserts such as cheesecake, crème caramel and custard (approximately twice a week), followed by ice-creams (1.5 times a week), ham (1.3 times a week) and industrial pastries (1.2 times a week) (Figure 7).

## 4. Discussion

The role of vitamin D in MS has been associated, among other functions, with an antioxidant activity by reducing oxidative stress levels [33]. In addition, along with its antioxidant function, vitamin D has strong anti-inflammatory and neuroprotective effects [34]. Specifically, it can suppress the expression and/or production of pro-inflammatory mediators and cytokines, IL-6 among them [34,35]. This is why hypovitaminosis D is shown to be an important environmental risk factor in MS [6,36]—considering the pathogenetic mechanisms of the disease—whereas high levels of vitamin D intake decrease the risk of suffering from the disease, improving prognosis due to its immunomodulatory and anti-inflammatory capacity [5,6]. This anti-inflammatory effect of vitamin D could be determined precisely by measuring this pro-inflammatory cytokine in blood and this is because, on the one hand, IL-6 has been directly linked to the pathogenesis of the disease [2,3], and, on the other hand, as previously mentioned, because vitamin D specifically decreases the levels of this cytokine [8], producing an immunomodulatory effect on the disease [9]. In this sense, our results confirm both premises, since IL-6 levels in patients are well above normal (1.4 pg/mL), as already seen in a previous study [37]. Furthermore, the intake of vitamin D seems relevant to IL-6 levels, confirmed by our results, since low intake of vitamin D significantly influences the increase in IL-6.

On the other hand, for people who do not suffer from the disease, and who also live in the Valencian Region. A study by Platero et al. 2020, carried out in our laboratory, showed that an isocaloric Mediterranean diet, typical of the Valencian region and characterized by high levels of antioxidants, decreased the levels of IL-6. Along these lines, it has been described by various authors that the Mediterranean diet contains a large amount of vitamin D [38,39].

Furthermore, in relation to the state of inflammation, it seems important not only to assess the vitamin levels but the origin. Thus, regarding the nutritional habits of this population related to vitamin D intake, it has been seen that a diet rich in omega-3 polyunsaturated fatty acids from blue fish, low in saturated animal fats, and supplemented with lipoic acid, has a beneficial effect on MS patients [40]. Therefore, the intake of vitamin D from unsaturated fats, and from a diet rich in proteins obtained mainly from blue fish, could be essential to improve the prognosis of MS.

Regarding proteins, our study shows that the vitamin content depends directly on protein rich foods, given the positive correlation between both variables. But at the same time, the main source of the protein ingested by the participants was from foods of animal origin, such as fortified milk, cured cheese and fortified yoghurts, reported by other authors to lead to increases in inflammation [41] and of IL-6 [42]. The participants also consumed little blue fish (such as tuna, mackerel, sardines or salmon), rich in proteins of high biological value, that according to Ciubotaru, et al. has shown an anti-inflammatory effect decreasing the levels of IL-6 in blood [43]. Moreover, in relation to the consumption of proteins, patients ingested few proteins of vegetable origin, which also, according to our regression analysis, significantly and negatively influences the high levels of IL-6. This is in line with what has been seen in another study that establishes the anti-inflammatory effect of the intake of proteins of vegetable origin [44]. Regarding fats, alterations in the nutritional habits of patients with MS have already been addressed by other authors, who highlighted the high intake of saturated fats of animal origin (mainly from red meat, dairy products and processed products) [18]. We specifically observe, since the World Health Organization (WHO) states that 20 to 35% of the calorie intake must be provided by fats, that 10.3% of the total fats consumed by the participants were saturated (when the recommended intake is less than 10%). These fats mainly came from foods of animal origin and processed foods (trans fats) such as industrial dairy desserts, salami, butter, industrial pastries and ice cream. It should be noted that saturated fats are related to poor absorption of vitamin D [45], especially when they come from processed foods [46], which would also help to explain the negative relationship between the intake of vitamin D and IL-6. Furthermore, the high levels of IL-6 in our population could be related to the fact that the levels of this interleukin decrease when the diet is based on the consumption of monounsaturated fats and not on the intake of saturated fats [47]. In this sense, and also regarding to the levels of inflammation, it is necessary to delve into the consumption of unsaturated fats in our population. We observe that the consumption of monosaturated fats is based on the intake of olive oil, while for the polyunsaturated, it is mainly due to the consumption of tuna and other fish. However, the percentages that they represented in the patients’ diets deviated from the recommendations, since the intake of monounsaturated fats represented 12.6% of the total fats, while the consumption of polyunsaturated fats represented 9.1%, and the WHO recommends a percentage of monounsaturated fats of around 15–20%, and polyunsaturated fats between 6–11% [48]. Hence, the consumption of monounsaturated fats should be much higher, as it has been seen that monounsaturated fats increase the absorption of vitamin D with respect to polyunsaturated fats [45]. We believe that this imbalance between both types of unsaturated fats could also contribute to the high levels of IL-6 determined in our study population.

Our study has limitations despite the results, which could help to understand the role of vitamin D in the pathogenesis of the disease based on the nutritional habits of the population with MS. On the one hand, we think that more in-depth biochemical analyses should be carried out, establishing blood levels of vitamin D in the form of its metabolites 25-hydroxyvitamin D or calcidiol, and mainly 1.25-dihydroxyvitamin D or calcitriol as the active metabolite of the vitamin, which would allow MS patients to be categorized according to their vitamin D status and then be compared with serum IL-6 levels. On the other hand, we believe that it is also important to replicate the study with a more representative population, since our work has sought to be an exploratory analysis of habits related to vitamin intake and of the state of inflammation of patients with MS. This should be confirmed with a larger population sample, from different geographic locations.

It is also necessary to consider variables such as smoking, which could affect IL-6 levels. Finally, it should be noted that in order to determine IL-6, plasma samples are more suitable than serum samples. Furthermore, the use of an immunofluorimetric assay would have been more sensitive and specific than ELISA.

## 5. Conclusions

Responding to the aims of the study, the analysis of our results seems to indicate that patients with MS in the Valencian region ingest little vitamin D, mainly from protein foods, more specifically of animal origin, rich in saturated fat and poor in vitamin, which could explain the inverse relationship with IL-6 levels as an inflammatory biomarker of MS.

## Figures and Tables

**Table 1:** Sociodemographic characteristics of patients with MS in the study group.

	Median (IQR)
Age (y)	47 (15)
EDSS	3.5 (3.9)
BMI (kg/m^2^)	23.88 (6.9)
Time from MS diagnosis (m)	12 (14)

EDSS: expanded disability status scale; BMI: body mass index; IQR: interquartile range; y: years; m: months.

**Table 2:** Regression coefficients for predictor variables in the multiple linear regression analysis for IL-6 blood levels in MS patients.

	b	t	*p*-Value	VIF
Constant	9.447	2.482	0.025	
Age (y)	0.01	0.261	0.798	1.155
MS type	−1.907	−1.986	0.064	1.633
EDSS	0.181	0.932	0.365	1.471
BMI (kg/m^2^)	−0.063	−0.868	0.398	1.385
Vitamin D (µg)	−0.437	−2.774	0.014	1.473
Animal Protein (g)	0.031	1.297	0.213	1.478
Vegetable Protein (g)	−0.123	−2.462	0.026	1.307

BMI: body mass index; b: regression coefficients; VIF: variance inflation factors; y: years; *p* < 0.05.
